# Integrated Microbiome and Metabolomic Analysis Reveal Responses of Rhizosphere Bacterial Communities and Root exudate Composition to Drought and Genotype in Rice (*Oryza sativa* L.)

**DOI:** 10.1186/s12284-023-00636-1

**Published:** 2023-04-11

**Authors:** Gege Li, Kexin Wang, Qun Qin, Qi Li, Fei Mo, Vinay Nangia, Yang Liu

**Affiliations:** 1 College of Agronomy, Northwest A&F University, Yangling, 712100 Shaanxi China; 2 International Center for Agricultural Research in the Dry Areas, 999055 Rabat, Morocco

**Keywords:** Rice, Drought, Genotype, Rhizosphere bacterial communities, Root exudates

## Abstract

**Background:**

As climate change events become more frequent, drought is an increasing threat to agricultural production and food security. Crop rhizosphere microbiome and root exudates are critical regulators for drought adaptation, yet our understanding on the rhizosphere bacterial communities and root exudate composition as affected by drought stress is far from complete. In this study, we performed 16S rRNA gene amplicon sequencing and widely targeted metabolomic analysis of rhizosphere soil and root exudates from two contrasting rice genotypes (Nipponbare and Luodao 998) exposed to drought stress.

**Results:**

A reduction in plant phenotypes was observed under drought, and the inhibition was greater for roots than for shoots. Additionally, drought exerted a negligible effect on the alpha diversity of rhizosphere bacterial communities, but obviously altered their composition. In particular, drought led to a significant enrichment of Actinobacteria but a decrease in Firmicutes. We also found that abscisic acid in root exudates was clearly higher under drought, whereas lower jasmonic acid and L-cystine concentrations. As for plant genotypes, variations in plant traits of the drought-tolerant genotype Luodao 998 after drought were smaller than those of Nipponbare. Interestingly, drought triggered an increase in *Bacillus*, as well as an upregulation of most organic acids and a downregulation of all amino acids in Luodao 998. Notably, both Procrustes analysis and Mantel test demonstrated that rhizosphere microbiome and root exudate metabolomic profiles were highly correlated. A number of differentially abundant genera responded to drought and genotype, including *Streptomyces*, *Bacillus* and some members of Actinobacteria, were significantly associated with organic acid and amino acid contents in root exudates. Further soil incubation experiments showed that *Streptomyces* was regulated by abscisic acid and jasmonic acid under drought.

**Conclusions:**

Our results reveal that both drought and genotype drive changes in the compositions of rice rhizosphere bacterial communities and root exudates under the greenhouse condition, and that organic acid exudation and suppression of amino acid exudation to select specific rhizosphere bacterial communities may be an important strategy for rice to cope with drought. These findings have important implications for improving the adaptability of rice to drought from the perspective of plant–microbe interactions.

**Supplementary Information:**

The online version contains supplementary material available at 10.1186/s12284-023-00636-1.

## Background

Global food security and crop production are greatly threatened by drought (Lesk et al. 2016; Mathobo et al. 2017; Zhao et al. 2021). The frequency, intensity and duration of drought events are predicted to increase due to global climate change, which further exacerbates the damage of drought on global agricultural production (Ault 2020). Therefore, extensive research has been conducted to explore plant responses and adaptations to drought to ensure agricultural productivity (de Vries et al. 2019; Gupta et al. 2020; Mu et al. 2021; Ghatak et al. 2022). Rice (*Oryza sativa* L.) is an important food crop, and its high and stable yields effectively insure higher global agricultural productivity. However, owing to the uneven spatial and temporal distribution of rainfall, rice is susceptible to drought stress (Liu et al. 2011). Drought significantly alters the physiological and metabolic processes of rice, ultimately reducing dry matter accumulation and grain yield (Yang et al. 2019). Additionally, different rice genotypes differ greatly in their responses to drought. For example, in a rice pot experiment, grain yields of the tolerant genotypes are less affected by drought than those of the sensitive genotypes (Wang et al. 2022a).

As a microecological region connecting plant roots and soil, the rhizosphere contains a large number of soil microbial communities that are involved in complex ecological and biological processes; hence, it is one of the most active interfaces in ecosystems (Tian et al. 2020). It has been reported that rhizosphere microbiome is closely related to host development (Lu et al. 2018; Chen et al. 2019; Li et al. 2021), nutrient absorption (Tao et al. 2019; Zhang et al. 2019) and pathogen immunity (Shi et al. 2019; Gu et al. 2020a; Tao et al. 2020). In particular, the role of rhizosphere microbiome in alleviating crop abiotic stresses has also attracted unprecedented attention in recent years (de Vries et al. 2020; Xu et al. 2021; Jin et al. 2022). Drought is probably the abiotic stress with the greatest impact on rhizosphere microbial communities (Leng and Hall 2019). A recent study has shown that drought alters nutrient availability and living environment of rhizosphere microbial communities, thereby affecting their diversity, composition and stability (Santos-Medellín et al. 2017; Jansson and Hofmockel 2020). These variations in rhizosphere microbial communities have the potential to affect soil carbon and nitrogen cycling, which can feed back into plant phenotypes under drought (de Vries et al. 2019). In addition, bacterial communities are more sensitive to drought than fungal communities (de Vries et al. 2018). Crop genotype is also responsible for some of the observed changes in rhizosphere microbial communities. However, knowledge about the effects of drought and different rice genotypes on the rhizosphere microbial communities is still limited.

Different parts of the plant root system exude or release a series of compounds into the rhizosphere—a process defined as root exudation (Shahzad et al. 2015; Williams and de Vries 2020). It is estimated that 5–21% of the carbon fixed by photosynthesis or 15–25% of the carbon allocated to the root system enters the soil as root exudates (Li et al. 2018). Root exudates consist predominantly of low-molecular-weight organic compounds such as amino acids, organic acids, sugars and secondary metabolites (Bais et al. 2006; Vives-Peris et al. 2020). In particular, amino acids and organic acids not only provide nitrogen and carbon sources for the growth and reproduction of rhizosphere microbial communities, respectively, but also act as chemotactic agents for specific microbial populations (Weisskopf et al. 2008; Gu et al. 2020b). Interest in root exudates has spiralled with the realization of their importance in crop responses to variations in the external environment (Chai and Schachtman 2022; Xiong et al. 2019). Drought can change the quantity and composition of root exudates (Calvo et al. 2017; Preece et al. 2018). Although some studies have focused on changes in root exudate quantity after drought, later studies have paid increasing attention to shifts in root exudate composition (Gargallo-Garriga et al. 2018; Bornø et al. 2022). It has been demonstrated that plants are able to actively modify root exudate composition and changes in the content of specific compounds in root exudates have an impact on the composition of rhizosphere microbial communities (Zhalnina et al. 2018). Furthermore, a growing number of researchers have found that the indirect effects via root exudates can outweigh the direct effects of drought on rhizosphere microbial communities (Preece and Peñuelas 2016). The composition of root exudates is also strongly dependent on plant genotype. For example, in a maize study, the concentration of fumaric acid in root exudates of drought-intolerant cultivars increases significantly under drought stress, while that of drought-tolerant cultivars has a minor variation (Song et al. 2012). Currently, metabolomic techniques have been utilized to explore the composition of root exudates. However, to the best of our knowledge, metabolome has rarely been used to examine the effects of drought and genotype on root exudate composition. The association between rhizosphere microbial communities and root exudates under different watering treatments and different genotypes has also not been investigated.

Therefore, rhizosphere soil samples and root exudates from two contrasting rice genotypes were collected at the end of a drought event in this study. The major objectives of this study were (1) to determine the effects of drought and genotype on the diversity and composition of rhizosphere bacterial communities using 16S rRNA gene amplicon sequencing and identify differentially abundant genera between different groups, (2) to conduct metabolomic profiling of root exudates from different watering treatments and two contrasting rice genotypes with widely targeted metabolomic analysis based on ultra performance liquid chromatography–tandem mass spectrometry (UPLC–MS/MS) and screen for differential metabolites in response to different conditions, and (3) to explore the associations between rhizosphere bacterial communities and root exudates. The results of this study can facilitate a deeper understanding of the differences in rhizosphere bacterial communities and root exudates in response to drought between two distinct rice genotypes, and provide new insights for improving the adaptation of rice production to drought from the perspective of plant–microbe interactions.

## Materials and Methods

### Experimental Design and Set-Up

A pot culture experiment was conducted in a greenhouse at Northwest A&F University, Yangling, Shaanxi, China (34.28 N and 108.07 E; 521 m a.s.l) in 2019. The soil used in the study was obtained in October 2018 from the upper 20 cm of a rice experimental field in Hanzhong, Shaanxi, China (33.18 N and 106.98 E; 548 m a.s.l). It was naturally air-dried and sieved through a 2 mm mesh. The soil contained 33.25 g kg^−1^ organic matter, 2.32 g kg^−1^ total nitrogen, 0.98 g kg^−1^ total phosphorus, 26.42 mg kg^−1^ available phosphorous, 19.77 g kg^−1^ total potassium, 181.00 mg kg^−1^ available potassium and a pH 5.62 at the beginning of the study.

Two contrasting rice genotypes were used in this study, of which Nipponbare was sensitive to drought and Luodao 998 was relatively drought tolerant. The rice seeds germinated and grew in seed trays using collected field soil in a light-temperature incubator. When the rice seedlings grew to the three-leaf and one-heart stage, 4 holes of consistently growing seedlings with 2 plants per hole were transplanted into each plastic bucket (height 19.5 cm, diameter 22.5 cm) containing 4 kg of field soil and receiving 1.62 g urea, 1.90 g NaH_2_PO_4_·2H_2_O and 1.44 g KCl before transplanting and grew to the five-leaf stage. For each genotype, 10 buckets were subsequently exposed to a 12-day drought treatment, and the others were fully watered. For the drought buckets, watering was stopped until a soil water potential of − 25 to − 35 kPa was reached, after which the buckets remained at this water potential until the end of drought, while the control buckets maintained shallow water layers of 1–2 cm throughout the treatment period. There were 10 replicates for each treatment, which resulted in a total of 40 buckets. All buckets were arranged in a completely randomized design in a greenhouse with a daily cycle of 30 °C during the day followed by 25 °C at night. The greenhouse was illuminated with natural light and supplemented with 600 μmol m^−2^ s^−1^ light for 2 h per day on cloudy or rainy days.

### Plant Sampling and Measurements

At the end of the simulated drought event, the plants were divided into shoots and roots. An EPSON 10000XL scanner was then employed to scan the roots, and the scanned images were analyzed using a LA-S root analyzer software (Wanshen Detection Technology Co., Hangzhou, Zhejiang, China) to obtain root morphological characteristics. After the analysis, the shoots and roots were dried at 80 °C for 72 h before weighing.

### Rhizosphere Soil Collection

Rhizosphere soil samples were collected according to the method described by Bulgarelli et al. (2012). Specifically, the aboveground plants were first removed. After the roots were gently shaken and kneaded with sterile gloves to shake off large chunks of soil, they were placed in sterile 50 mL tubes with 25 mL phosphate buffer, which was prepared from 130 mM NaCl, 7 mM Na_2_HPO_4_, 3 mM NaH_2_PO_4_ and 0.02% Silwet L-77. Following vortexing at maximum speed for 15 s, the roots were transferred with sterilized tweezers to another sterile 50 mL tube containing 25 mL phosphate buffer and continued to vortex for 15 s. Subsequently, the collected soil suspension was filtered through a 100-µm nylon mesh cell strainer into a new 50 mL tube and centrifuged at 10,000 rpm for 15 min to form fine sediment particles; these were defined as the rhizosphere soils. Finally, the rhizosphere soils were snap-frozen in liquid nitrogen and stored at − 80 °C until later high-throughput sequencing.

### Soil Incubation Experiment

To further determine whether root exudates have the potential to select specific bacterial communities in the rhizosphere under drought stress, an additional soil incubation experiment was performed. Abscisic acid and jasmonic acid (0.2 mM), two organic acids identified in both rice genotypes in response to drought, and a sterile ultrapure water control were added to 30 g of soil placed in a sterile glass bottle. The soil used in the incubation experiment was the same as that used in the greenhouse experiment. Before drought stress treatment, the glass bottles were pre-incubated for 1 week by maintaining a shallow water layer of 1–2 cm in an incubator with a daily dark cycle of 30 °C for 14 h followed by 25 °C for 10 h. Each glass bottle was then subjected to drought stress at a soil water potential of − 25 to − 35 kPa, while receiving 2 mL of additives adjusted to a neutral pH (7.0) per day. After 12 days, soil samples were collected for 16S high-throughput sequencing. Each treatment was incubated in triplicate.

### DNA Extraction, Illumina Sequencing and Analysis

Total soil genomic DNA was extracted from the collected soil (0.5 g) using the FastDNA™ SPIN Kit for Soil (MP Biomedicals, Santa Ana, CA, USA) following the manufacturer’s instructions, and the DNA concentration and quality were then determined with a NanoDrop 2000 spectrophotometer (Thermo Scientific, Wilmington, DE, USA) and 1% agarose gel electrophoresis, respectively. The V3–V4 hypervariable region of the bacterial 16S rRNA gene was amplified with the primer pair 338F (5′-ACT CCT ACG GGA GGC AGC AG-3′) and 806R (5′-GGA CTA CHV GGG TWT CTA AT-3′) using a PCR thermocycler (ABI GeneAmp 9700, Foster City, CA, USA) (Peiffer et al. 2013). Equimolar amounts of purified PCR products from each DNA sample were pooled and sequenced using the Illumina MiSeq PE300 platform (San Diego, CA, USA) at Majorbio Bio Pharm Technology Co., Ltd. (Shanghai, China). The detailed protocols for PCR thermal cycling and 16S amplicon sequencing have been described previously (Caporaso et al. 2010).

The raw 16S rRNA sequences were quality filtered and trimmed with fastp version 0.19.6 (Chen et al. 2018) and merged using FLASH (Reyon et al. 2012). USEARCH was used to remove chimeras to obtain high-quality sequences (Edgar 2010). High-quality sequences were assigned to each soil sample by specific barcodes using QIIME (Caporaso et al. 2010) and then clustered into operational taxonomic units (OTUs) at a 97% similarity level using UPARSE pipeline (Edgar 2013). Each OTU was annotated with different levels of taxonomic information using the RDP Classifier (Wang et al. 2007) against the SILVA 16S rRNA database (Release 138, https://www.arb-silva.de). A total of 486,287 and 218,816 high-quality sequences were generated in the greenhouse experiment (34,700–45,515 per sample, with a median of 40,119) and soil incubation experiment (21,940–26,321 per sample, with a median of 24,393), respectively. Both rarefaction curves revealed that the sequencing depth of bacterial communities had reached saturation (Additional file 2: Fig. S1). Each soil sample was rarefied to the minimum sequence for downstream diversity analysis.

### Root Exudate Collection

Root exudates were collected using the method described by de Vries et al. (2019). Briefly, the roots of intact plants were carefully washed to remove any remaining soil, during which time the roots were left as undamaged as possible, and dead roots were removed with stainless steel tweezers. One hole of rice plants was then transferred to 100 mL soil solutions made up of 1:10 in situ soil:Milli-Q water and incubated for 12 h in situ greenhouse conditions. After 12 h of incubation, the rice roots were rinsed and transferred to conical flasks containing 100 mL sterilized Milli-Q water placed on ice. Additionally, the conical flasks were shaken at 60 rpm for 2 h at 30 °C and ambient light, and the liquid collected in the conical flasks was regarded as root exudate. A 30 mL sample of each root exudate solution was filtered through a 0.22 μm millipore filter membrane to remove any root debris, flash-frozen in liquid nitrogen and then stored at − 80 °C until further metabolomic analysis.

### Metabolomic Analysis of Root Exudates

Metabolomic profiling of root exudates was conducted using an ultra performance liquid chromatography system (UPLC, SHIMADZU Nexera X2, Kyoto, Japan) with tandem mass spectrometry (MS/MS, Applied Biosystems 4500 QTRAP, Framingham, MA, USA). First, root exudates were removed from − 80 °C, thawed on ice and vortexed for 10 s to mix. A total of 10 mL of the mixed sample was transferred to a 15 mL centrifuge tube, quickly frozen in liquid nitrogen and lyophilized in a vacuum freeze-dryer. When all samples were completely lyophilized to powder, 300 μL of 70% methanol internal standard extract was added to the sample. Following vortexing for 3 min and centrifugation (12,000 rpm, 4 °C) for 10 min, the supernatants were transferred to sample bottles for subsequent analysis. Each sample was mixed in equal amounts as a quality control (QC) sample. When root exudate samples were analyzed using an UPLC–MS/MS, one QC sample was inserted for every 10 samples. The detailed conditions for UPLC and MS/MS were set according to Wang et al. (2022b). The primary metabolites in the root exudates were identified using the Metware database (Metware Biotechnology Co., Ltd., Wuhan, China) and quantified by multiple reaction monitoring of triple quadrupole mass spectrometry.

### Statistical Analysis

SPSS 23.0 (SPSS Inc., Chicago, IL, USA) and R software (version 4.1.1) were used to conduct all statistical analyses. Two-way analysis of variance (ANOVA) followed by least significant difference (LSD) test was done to test the significance of the effects of drought and genotype on plant phenotypes, alpha diversity indices of rhizosphere bacterial communities and relative abundances of dominant phyla using SPSS 23.0.

Principal coordinate analysis (PCoA) based on Bray–Curtis distance was calculated and plotted, using the R package vegan, to determine the main variable components in the rhizosphere bacterial communities (Dixon 2003). Significant tests of various experimental factors and their interactions on bacterial community composition were performed using permutational multivariate analysis of variance (PERMANOVA) with 999 permutations based on Bray–Curtis distance. Differentially abundant genera in response to different watering treatments and the two rice genotypes were generated by the linear discriminant analysis (LDA) using the linear discriminant analysis effect size (LEfSe) method (Segata et al. 2011). Venn diagrams showing shared and specific genera between different groups were also constructed.

Hierarchical cluster analysis and Pearson’s correlation analysis were used to examine the intra-treatment homogeneity of root exudate. The PCoA of primary metabolites based on Bray–Curtis distance was built using the R package vegan. PERMANOVA based on 999 permutations using Bray–Curtis distance was done to analyze the effects of drought and genotype on root exudate composition. Orthogonal partial least squares discriminant analysis (OPLS-DA) was conducted in the R ropls package, and the models were further evaluated with 200 permutations (Thévenot 2021). Metabolites with (1) variable importance in the projection (VIP) ≥ 1, which was extracted from the OPLS-DA result, and (2) fold change ≥ 2 and fold change ≤ 0.5 were regarded as differential metabolites. Volcano plots were generated using the ggpubr package in R to screen for differential metabolites between the different watering treatments and between the two rice genotypes (Kassambara 2022). To identify shared and exclusive differential metabolites between different groups, Venn diagrams were constructed using the R package venndiagram (Chen and Boutros 2011).

To examine the correlation between rhizosphere bacterial communities and root exudates, a Procrustes analysis based on the PCoA results of the abundance of all genera and all identified metabolites was performed. Meanwhile, a Mantel test using the Spearman correlation method was conducted between them. Spearman’s correlation analysis was further used to assess the associations between differentially abundant genera and differential metabolites using the corrplot package in R (Wei and Simko 2017). All *p*-values in the Spearman’s correlation were adjusted by Benjamini and Hochberg using the false discovery rate (FDR) control procedure (Benjamini and Hochberg 1995).

## Results

### Effects of Drought and Genotype on Plant Phenotypes

**Fig. 1:**
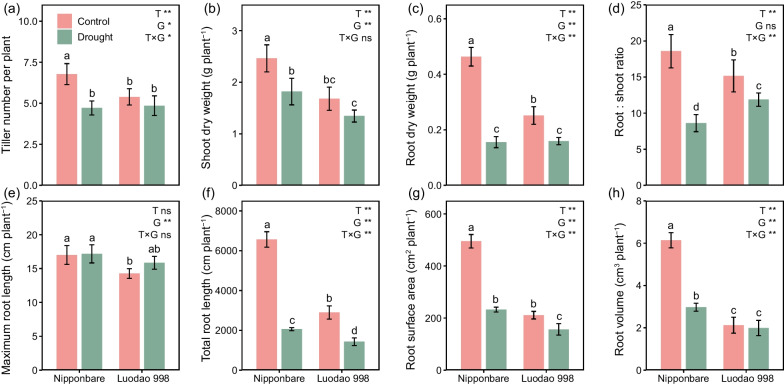
**a** Tiller number, **b** shoot dry weight, **c** root dry weight, **d** root: shoot ratio, and **e**–**h** root traits as affected by drought and genotype. Error bars represent standard deviation (n = 4). Different letters (*p* < 0.05) and asterisks (*p < 0.05 and **p < 0.01) indicate significant differences as defined by two-way ANOVA with LSD test. “ns” means non-significant difference. *T* Treatment, *G* Genotype

Tillers, dry matter weights and root traits were significantly affected by drought, except for maximum root length (Fig. 1). In both Nipponbare and Luodao 998, drought considerably reduced tillers, shoot dry weight, root dry weight and the ratio of root-to-shoot dry weight (Fig. 1a–d). Notably, the reduction in root dry weight was stronger than that in shoot dry weight. Additionally, total root length, root surface area and root volume were all at least 1.7 times higher under well-watered conditions than under drought conditions (Fig. 1f–h).

The plant properties of the two rice genotypes displayed great differences. On average, the tillers, shoot dry weight and root dry weight of Nipponbare were 1.12-, 1.42- and 1.50-fold greater than those of Luodao 998, respectively (Fig. 1a–c). In particular, the reductions in these properties (except shoot dry weight) and the ratio of root-to-shoot dry weight under drought were more pronounced in Nipponbare. The two rice genotypes also differed greatly in their root morphology, with Nipponbare having higher maximum root length, total root length, root surface area and root volume (Fig. 1e–h). Similarly, drought decreased total root length, root surface area and root volume of Nipponbare more than those of Luodao 998.

### Effects of Drought and Genotype on Rhizosphere Bacterial Communities

**Fig. 2:**
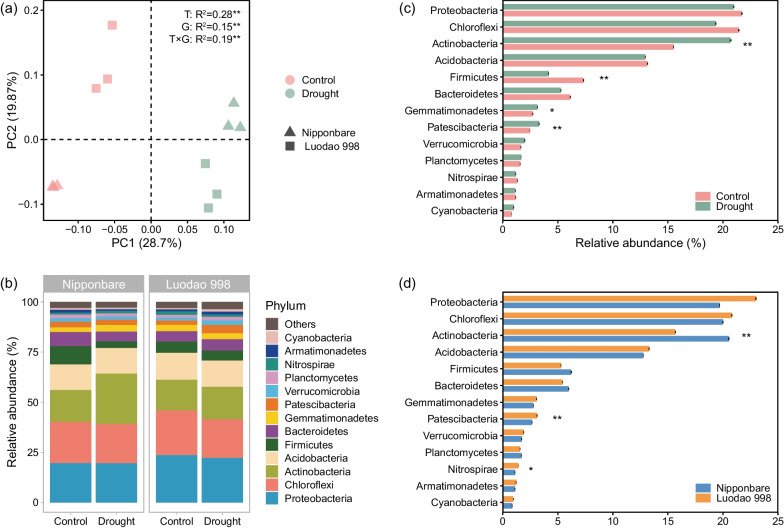
Structure and taxonomic information of rhizosphere bacterial communities as affected by drought and genotype. **a** Principal coordinates analysis (PCoA) of rhizosphere bacterial communities based on Bray–Curtis distance at the OTU level. Asterisks indicate significant differences as defined by PERMANOVA (**p < 0.01). *T* Treatment, *G* Genotype. **b** Relative abundance of the dominated bacterial phyla in rhizosphere soil samples. Bar graphs showing the distribution of dominated phyla of rhizosphere bacterial communities **c** between the different watering treatments and **d** between the two rice genotypes. Asterisks indicate significant differences as defined by two-way ANOVA with LSD test (*p < 0.05 and **p < 0.01)

The alpha diversity of the rhizosphere bacterial communities was not regulated by drought (Additional file 1: Table S1). The Sobs index and Chao 1 index of the rhizosphere bacterial communities of the two rice genotypes were similar. However, Luodao 998 had higher Shannon index. PCoA ordinations and PERMANOVA analyses were employed to determine the effects of various experimental factors and their interactions on composition of rhizosphere bacterial communities. Our results demonstrated that watering treatment was the main driver of rhizosphere bacterial community composition (R^2^ = 0.28, *p* = 0.001). The impacts of genotype (R^2^ = 0.15, *p* = 0.002) and interaction effect (R^2^ = 0.19, *p* = 0.001) were also significant (Fig. 2a).

According to taxonomic classification, Proteobacteria (19.65–23.69%), Chloroflexi (19.23–22.37%) and Actinobacteria (15.17–25.18%) were dominant in all rhizosphere soil samples at the phylum level (Fig. 2b). Bar graphs for each taxon further revealed that after a period of drought, the relative abundance of Actinobacteria, Gemmatimonadetes and Patescibacteria increased by 33%, 16% and 34%, respectively, while that of Firmicutes decreased by 43% (Fig. 2c and Additional file 1: Table S2). In addition, Actinobacteria was dramatically depleted in Luodao 998, whereas Patescibacteria and Nitrospirae were highly enriched in Luodao 998 (Fig. 2d and Additional file 1: Table S2).

**Fig. 3:**
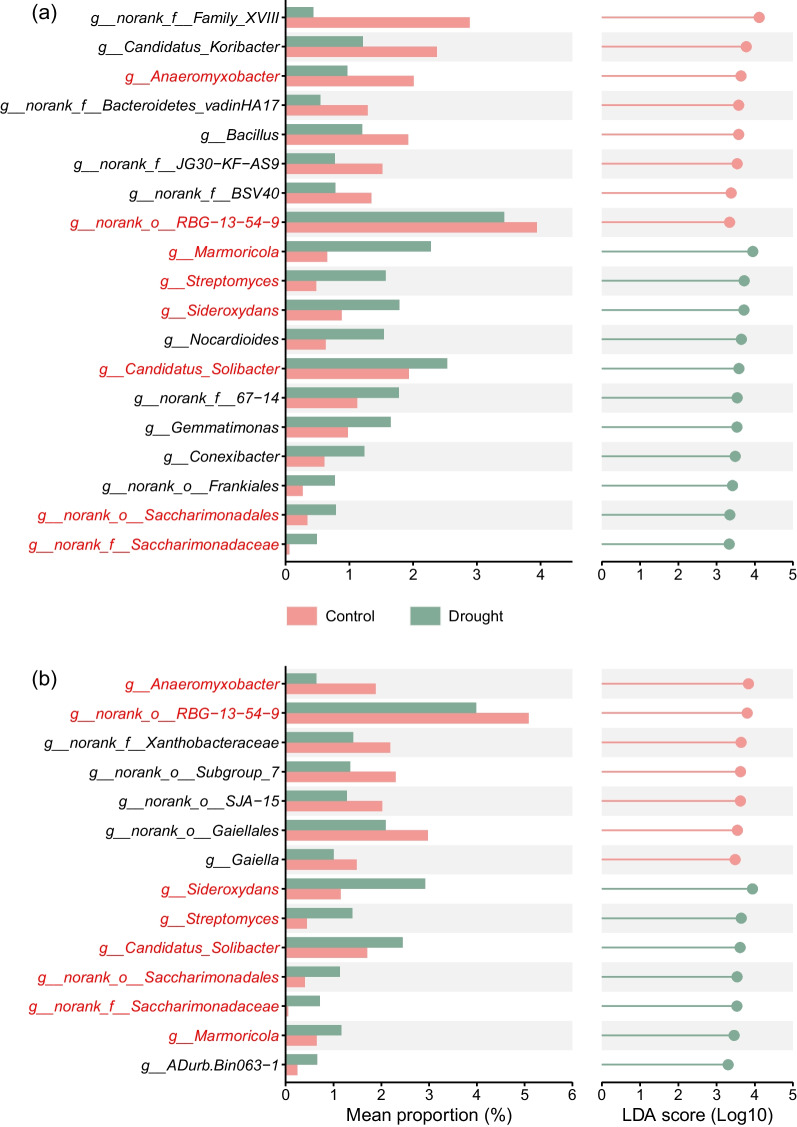
Differentially abundant genera identified by LEfSe with logarithmic LDA > 3.3 in response to different watering treatments in **a** Nipponbare and **b** Luodao 998. The abscissa on the left represents the mean relative abundance of a certain genus under different treatments, and the ordinate represents the name of the genus. The name of the genus marked in red denotes that the genus is shared by the two rice genotypes. The length of the line on the right reflects the LDA value, and the color of the nodes and lines indicates that the genus is significantly enriched in the corresponding watering treatment

**Fig. 4:**
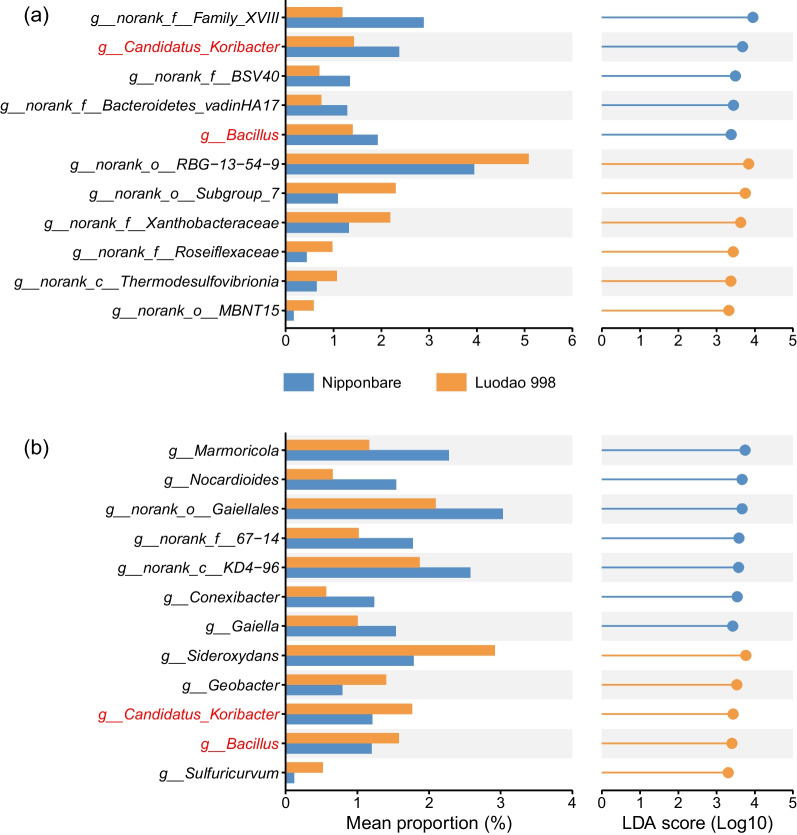
Differentially abundant genera identified by LEfSe with logarithmic LDA > 3.3 in response to the two rice genotypes under **a** control and **b** drought conditions. The abscissa on the left represents the mean relative abundance of a certain genus in two distinct rice genotypes, and the ordinate represents the name of the genus. The name of the genus marked in red denotes that the genus is shared by the control and drought groups. The length of the line on the right reflects the LDA value, and the color of the nodes and lines indicates that the genus is significantly enriched in the corresponding rice genotype

LEfSe showed that 19 differentially abundant genera were identified between different watering treatments in Nipponbare and 14 in Luodao 998 (Fig. 3). In particular, a total of 8 genera overlapped between the two genotypes (Additional file 2: Fig. S2a). The relative abundance of *Marmoricola*, *Streptomyces*, *Sideroxydans* and *Candidatus_Solibacter* was greater under drought for both rice genotypes, while that of *Anaeromyxobacter* was lower in the drought treatment (Fig. 3). Meanwhile, 11 and 12 key genera were differently abundant between the two rice genotypes under control and drought conditions, respectively (Fig. 4). Notably, *Bacillus* and *Candidatus_Koribacter* were elevated in Nipponbare in control group, while they were overabundant in Luodao 998 in water deprivation.

### Effects of Drought and Genotype on Root Exudate Composition

To investigate the responses of root exudate composition to drought and genotype, widely targeted metabolomic analysis using UPLC–MS/MS was carried out on root exudate samples. In total, 269 metabolites were detected at level 1, including 47 amino acids and their derivatives, 35 organic acids, 37 phenolic acids, 32 nucleotides and derivatives, 70 lipids and 48 other compounds (Additional file 2: Fig. S3). The three replicates of each group were highly correlated, as also evidenced by hierarchical cluster analysis, which confirmed the homogeneity of the root exudate samples (Additional file 2: Figs. S3 and S4). The PCoA exhibited a clear separation between control and drought samples in Luodao 998 on the first axis (Additional file 2: Fig. S5). The PERMANOVA analyses further demonstrated that watering treatment explained the greatest variation in root exudates (R^2^ = 0.31, *p* = 0.001), followed by genotype (R^2^ = 0.28, *p* = 0.001) and interaction effect (R^2^ = 0.21, *p* = 0.001) (Additional file 1: Table S3).

**Fig. 5:**
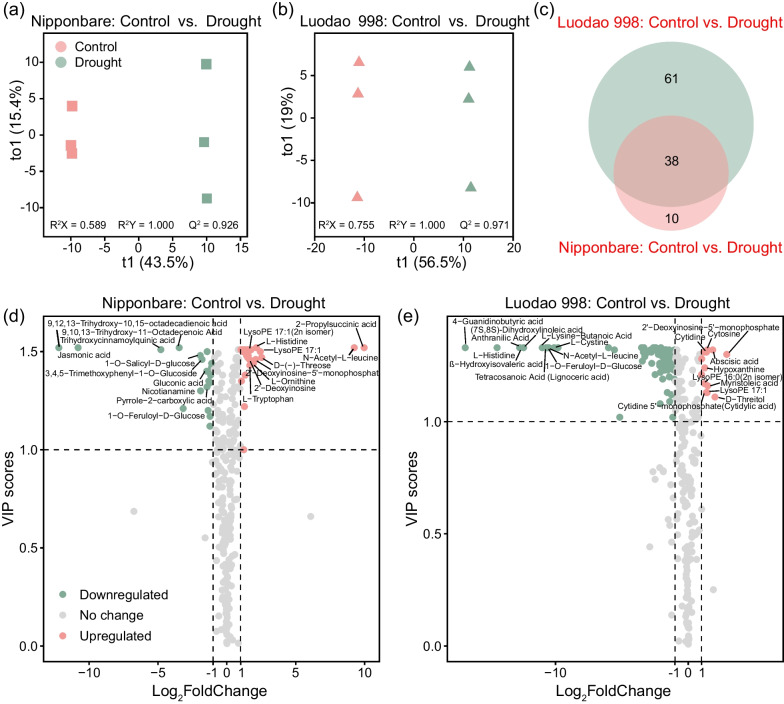
Changes in root exudate composition between different watering treatments. (**a**, **b**) Metabolomic profiles of root exudates between control and drought conditions in the two rice genotypes assessed by OPLS-DA. **c** Number of differential metabolites between different watering groups in the two rice genotypes. Volcano plots of differential metabolites between control and drought treatments in **d** Nipponbare and **e** Luodao 998. The top 10 differential metabolites with the largest and smallest fold changes are labelled with text

According to the above findings, OPLS-DA was first used to analyze the differences in root exudates between the control and drought groups in the two contrasting genotypes. The results revealed that the control and drought samples were clearly separated in both genotypes (Fig. 5a, b). Moreover, we constructed volcano plots using fold change and VIP to screen for differential metabolites between the control and drought conditions. And 48 differential metabolites were identified following different watering treatments for Nipponbare and 99 for Luodao 998 (Fig. 5d, e). Among them, 38 metabolites were shared between the two rice genotypes (Fig. 5c). The relative content of abscisic acid consistently increased during water deficit in the two genotypes (Additional file 2: Fig. S6a). Reductions in L-cystine and several organic acids, including jasmonic acid, pyrrole-2-carboxylic acid, D-xylonic acid and 9-oxononanoic acid, were distinctly observed in both genotypes (Additional file 2: Fig. S6b–f).

**Fig. 6:**
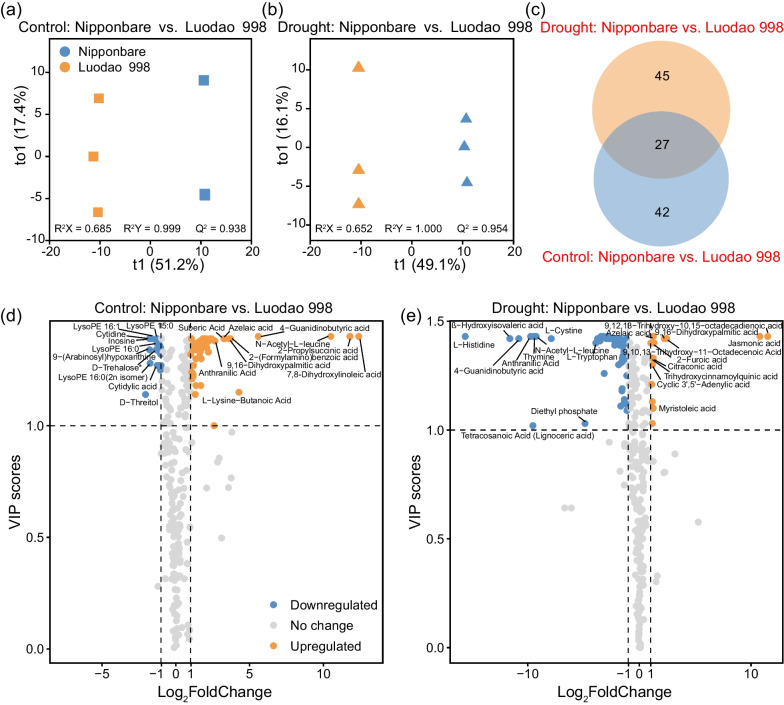
Changes in root exudate composition between the two rice genotypes. Metabolomic profiles of root exudates between the two contrasting rice genotypes under **a** control and **b** drought conditions assessed by OPLS-DA. **c** Number of differential metabolites between the two rice genotypes under different watering treatments. Volcano plots of differential metabolites between the two distinct rice genotypes in **d** control and **e** drought groups. The top 10 differential metabolites with the largest and smallest fold changes are labelled with text

Similarly, to distinguish genotypic differences in control and drought conditions independently, we ran OPLS-DA on root exudate samples. Our results showed that the metabolic composition of Nipponbare deviated obviously from that of Luodao 998 regardless of the watering treatments (Fig. 6a, b). Additionally, volcano plots were used to visualize differential metabolites between the two rice genotypes, in which 69 and 72 differential metabolites were detected in the control and drought groups, respectively (Fig. 6d, e). In particular, most organic acids, including jasmonic acid, 2-furoic acid, citraconic acid, azelaic acid and suberic acid, exhibited upregulated properties in Luodao 998 under drought stress (Additional file 2: Fig. S7a–e). However, downregulated levels of all amino acids such as L-histidine, L-cystine, L-leucine and L-proline were found in Luodao 998 under drought conditions (Additional file 2: Fig. S7f–i).

### Associations between rhizosphere bacterial communities and root exudates

**Fig. 7:**
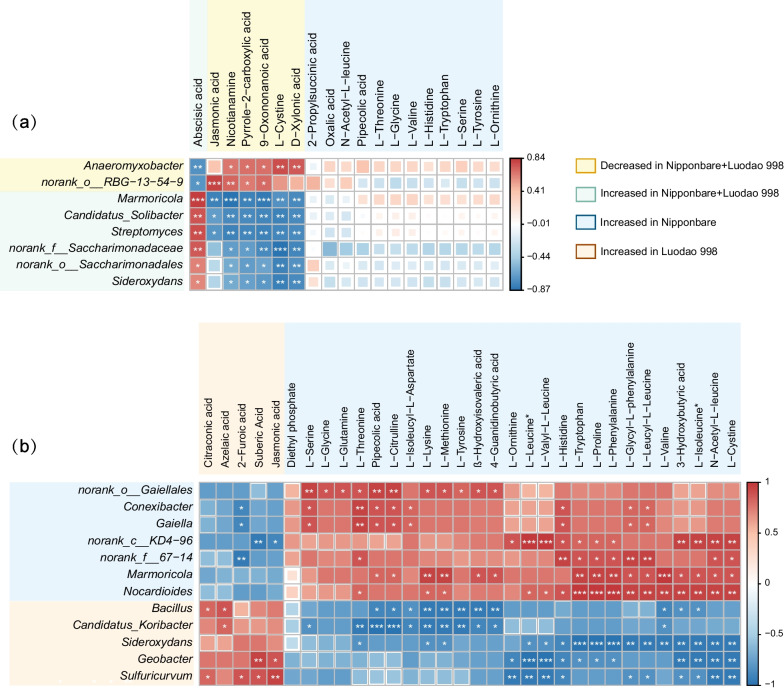
Associations between rhizosphere bacterial communities and root exudates. Correlation analyses of differentially abundant genera and differential root exudates **a** for both rice genotypes and **b** under drought conditions. The abundance of genera and metabolites is visualized after log2 transformation. Asterisks indicate significant correlations (*FDR < 0.05, **FDR < 0.01 and ***FDR < 0.001; Spearman correlation)

Rhizosphere soil microbiome and root exudate metabolomic profiles were highly correlated across all samples (Procrustes analysis, M^2^ = 0.2554, *p* = 0.001; Mantel test, r = 0.2780, *p* = 0.024), confirming the strong links between rhizosphere bacterial communities and root exudates (Additional file 2: Fig. S8). Subsequently, we performed Spearman’s correlation analyses to determine the associations between differentially abundant genera and differential metabolites. In general, we found strong positive correlations between genera and metabolites that were both increased or decreased in the same condition, as well as high negative correlations between elevated genera and depleted metabolites (Fig. 7).

Notably, *Streptomyces* displayed a positive correlation with abscisic acid (r = 0.797, FDR = 0.002) and a negative correlation with jasmonic acid (r = − 0.697, FDR = 0.012) (Additional file 2: Fig. S9a–b). We also observed that *Conexibacter*, *Gaiella*, *Marmoricola* and *Nocardioides*, some members of Actinobacteria, were positively associated with L-threonine (r = 0.943, FDR = 0.005), L-threonine (r = 0.943, FDR = 0.005), L-valine (r = 1.000, FDR = 0.000) and L-tryptophan (r = 1.000, FDR = 0.000), respectively (Additional file 2: Fig. S9c–f). Additionally, *Bacillus*, a plant growth-promoting rhizobacterium elevated in Luodao 998 under drought, was positively associated with azelaic acid (r = 0.886, FDR = 0.019) and citraconic acid (r = 0.829, FDR = 0.042) (Additional file 2: Fig. S9g–h).

**Fig. 8:**
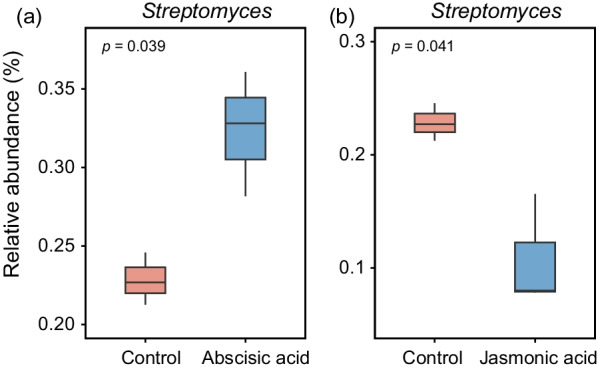
Changes in relative abundance of specific bacterial genera following the addition of selected **a** abscisic acid and **b** jasmonic acid under drought stress. The center lines of the boxes indicate the medians, the top and bottom of the boxes indicate the first and third quartiles, and the whiskers indicate the 1.5 interquartile range. The *p*-values are calculated by t-test

To establish more causal relationships between root exudates and rhizosphere bacterial communities under drought stress, an additional soil incubation experiment was conducted. After a 12-day drought event, the relative abundance of *Streptomyces* in soil added with abscisic acid was higher than in soil added with sterile water (Fig. 8a). However, comparative analysis of bacterial communities at the genus level between jasmonic acid addition and control under drought stress showed that jasmonic acid significantly suppressed the relative abundance of *Streptomyces* (Fig. 8b).

## Discussion

Rhizosphere microbiome and root exudates are critical in crop response or adaptation to drought, and their differential responses are closely related to genotype (Calvo et al. 2017; Gargallo-Garriga et al. 2018; Santos-Medellín et al. 2021; Xie et al. 2021). However, less attention has been paid to determining the effects of drought and genotype on the diversity and composition of rhizosphere bacterial communities and root exudate composition. In this study, using 16S rRNA gene amplicon sequencing and widely targeted metabolomic analysis based on UPLC–MS/MS, we explored the shifts in plant traits, rhizosphere bacterial communities and root exudates as affected by drought stress and genotype, identified differentially abundant genera and differential metabolites between different conditions and investigated associations between rhizosphere bacterial communities and root exudates.

### Plant Phenotypes, Rhizosphere Bacterial Communities and Root Exudates in Response to Drought

The production, accumulation and distribution of dry matter are the bases for rice yield formation. Drought can affect dry matter production and accumulation in rice (Kumar et al. 2006). In this study, both shoot dry weight and root dry weight of rice under drought were significantly lower than those under well-watered conditions (Fig. 1b, c). Moreover, the root dry weight was more inhibited by drought than the shoot dry weight, which resulted in a significant decrease in the ratio of root-to-shoot dry weight. This result is consistent with previous studies showing that root growth is more sensitive to water deficit than shoot growth (de Vries et al. 2019). It was further confirmed by the fact that root morphology, such as total root length, root surface area and root volume, was greatly reduced under drought (Fig. 1f–h).

The diversity and composition of soil microbial communities can respond to drought (Preece et al. 2019). It is generally believed that the higher the rhizosphere microbial diversity, the stronger the stability of the soil ecosystem and the stronger the resistance to drought (Xun et al. 2021). In our study, drought had a negligible effect on the alpha diversity of rhizosphere bacterial communities (Additional file 1: Table S1). This finding is supported by previous studies suggesting that drought does not directly lead to the death of drought-sensitive bacterial communities and the emergence of drought-tolerant bacterial communities in the rhizosphere, but rather keeps the diversity of rhizosphere bacterial communities relatively stable (Bastida et al. 2017; Tóth et al. 2017; Xie et al. 2021). In contrast to alpha diversity, drought dramatically altered the composition of rhizosphere bacterial communities. Our results indicated that Actinobacteria, Gemmatimonadetes and Patescibacteria were highly enriched in drought (Fig. 2c and Additional file 1: Table S2). Notably, Actinobacteria, a Gram-positive bacterium, is able to exploit carbon sources that are difficult to decompose, so it is abundant in nutrient-poor but oxygen-rich arid environments (Yuste et al. 2014; Mohammadipanah and Wink 2016). It also has a thicker cell wall that can better resist water stress (Lennon et al. 2012; Xu and Coleman-Derr 2019). In addition, several members of Actinobacteria possess filamentous growth habits and the abilities to produce spores, which enables them to enter dormant states under environmental stress (Barka et al. 2016). These properties may explain the prominent enrichment of Actinobacteria in the rhizosphere soil under drought conditions.

Changes in root exudate composition under drought have crucial implications for the plants themselves, soil properties, and especially soil microbial communities. In the present study, widely targeted metabolomic techniques based on UPLC–MS/MS were employed to investigate the response of root exudate composition to drought. These techniques can provide comprehensive biochemical information on the metabolism involved in the biosynthesis of primary metabolites in root exudates. We observed that the drought and control samples were separated into two distinct groups regardless of the rice genotype (Fig. 5a, b). Further differential metabolite analysis revealed that some compounds, such as jasmonic acid and abscisic acid, were strongly affected by drought in both rice genotypes (Additional file 2: Fig. S6a, b). Previous studies have implied that an elevated abundance of *Streptomyces* is found in rhizosphere soil samples of JA signalling-compromised Arabidopsis mutants (Carvalhais et al. 2015). Also in our results, the addition of jasmonic acid significantly reduced the relative abundance of *Streptomyces* under drought stress, which further confirmed the above studies (Fig. 8b). Intriguingly, we observed an upregulation of abscisic acid in root exudate samples under drought. This result is in agreement with recent studies suggesting that abscisic acid in root exudates is strongly induced during drought (Yang et al. 2002; Calvo et al. 2017; Gargallo-Garriga et al. 2018). Moreover, addition of abscisic acid enriched *Streptomyces* under drought stress, highlighting the involvement of abscisic acid in helping plants shape rhizosphere microbial communities under abiotic stress (Belimov et al. 2014).

### Plant Phenotypes, Rhizosphere Bacterial Communities and Root Exudates in Response to Two Distinct Genotypes

Changes in plant properties under abiotic stress are highly dependent on plant genotype. As demonstrated in our results, the variations in plant traits under drought were more pronounced in Nipponbare than in Luodao 998 (Fig. 1). Previous researchers have found similar results (Ji et al. 2012). These findings reveal that tolerant genotypes can reduce the damage of drought stress on dry matter production and root morphology to maintain plant growth and development.

Plant selectivity is essential for the establishment of soil microbial communities under adverse conditions. At the genus level, drought led to an increase in the relative abundance of *Bacillus* in Luodao 998 (Fig. 4b). *Bacillus*, a plant growth-promoting rhizobacterium, has been reported to improve drought tolerance in many plant species (Wang et al. 2019; Xie et al. 2019; Fonseca et al. 2022; Kim et al. 2022). For example, *Bacillus* enhances antioxidant enzyme activity and photosynthetic efficiency in wheat, which may be a cause of drought tolerance (Rashid et al. 2022). *Bacillus* also regulates auxin levels to protect wheat from drought stress (Barnawal et al. 2017). Additionally, inoculation of *Bacillus* in maize activates the exudation of osmotic substances such as proline, amino acids and soluble sugars to promote plant survival under drought conditions (Vardharajula et al. 2011). These findings highlight that the tolerant genotype can mitigate severe drought damage to plants by recruiting plant growth-promoting rhizobacteria.

It has been reported that host can affect the composition of root exudates (Ghatak et al. 2022). Using a PCoA, we found that drought induced greater changes in the composition of root exudates in Luodao 998 compared to Nipponbare (Additional file 2: Fig. S5). This differential response is attributed to the fact that drought can stimulate the physiological and metabolic activities of root systems of tolerant genotypes to alleviate negative effects on plants in the short term. For specific root exudate components, under drought stress, the vast majority of metabolites, especially all amino acids, displayed downregulated properties in Luodao 998 compared with Nipponbare (Fig. 6e). We speculated that drought triggered the accumulation of amino acids in roots of the tolerant genotype, thereby increasing resistance to drought. This accumulation is believed to strengthen plant stress resistance by affecting physiological mechanisms such as adjustment of osmotic changes, ROS detoxification and regulation of intracellular pH levels (Good and Zaplachinski 1994). In a previous study, the accumulation is also conducive to maintaining root development for accessing deeper water sources (Serraj and Sinclair 2002). It is likely that the accumulation effect contributes to reducing drought damage to the roots of Luodao 998. Moreover, our data also indicated that under drought stress, amino acids in root exudates correlated positively with some members of Actinobacteria, including *Conexibacter*, *Gaiella*, *Marmoricola* and *Nocardioides* (Fig. 7b and Additional file 2: Fig. S9c–f), which was supported by earlier studies showing that amino acid addition can enrich Actinobacteria (Eilers et al. 2010; Gu et al. 2020b). Therefore, the decline of Actinobacteria members in Luodao 998 under drought may be explained by a reduction in amino acid exudation. Although the vast majority of metabolites were downregulated in Luodao 998 at the end of drought, most organic acids were upregulated (Additional file 2: Fig. S7). It is known that drought causes a decrease in the availability of phosphorus in soil (Sardans and Peñuelas 2004). Drought-mediated accumulation of organic acids in the drought-tolerant genotype helps solubilize unavailable phosphorus from soil (Li et al. 2007; Khademi et al. 2010; Pantigoso et al. 2020; Bi et al. 2021). In addition, some organic acids are potent chemotactic agents for *Bacillus*, which is responsible for the enrichment of *Bacillus* in the tolerant genotype (Allard-Massicotte et al. 2016).

## Conclusions

Integrated microbiome and metabolomic analysis revealed that both drought and genotype had significant effects on the compositions of rhizosphere bacterial communities and root exudates under the greenhouse condition. Notably, a strong link was observed between various differentially abundant genera and levels of organic acids and amino acids in root exudates, and the abundance of specific bacterial genera was regulated by the identified differential root exudates under drought stress, suggesting that organic acid exudation and suppression of amino acid exudation to select specific rhizosphere bacterial communities might be an important strategy for rice to respond to drought stress.

## Supplementary Information

**Additional file 1**: **Table S1**. Effects of drought and genotype on alpha diversity of rhizosphere bacterial communities. **Table S2**. Effects of drought and genotype on relative abundances (%) of the dominated bacterial phyla. **Table S3**. Significant tests of drought and genotype on metabolomic properties of root exudates.

**Additional file 2**: **Fig. S1**. Rarefaction curves of bacterial communities for the **a** greenhouse experiment and **b** soil incubation experiment. **Fig. S2**. Number of differentially abundant genera **a** between the two rice genotypes and **b** between the different watering treatments. **Fig. S3**. Hierarchical cluster analysis of primary metabolites identified in four groups. **Fig. S4**. Correlation analysis of all identified metabolites for each root exudate sample. **Fig. S5**. Principal coordinates analysis (PCoA) of root exudates based on Bray–Curtis distance. **Fig. S6**. Representative differential metabolites between control and drought treatments for both rice genotypes. **Fig. S7**. Representative differential metabolites between Nipponbare and Luodao 998 under drought. **Fig. S8**. Procrustes analysis and Mantel test of the correlation between rhizosphere bacterial communities and root exudates. **Fig. S9**. Examples of associations between individual differentially abundant genera and differential root exudates.

## Data Availability

The raw 16S rRNA reads have been uploaded to the NCBI Sequence Read Archive (SRA) database with the accession number PRJNA907252.

## Abbreviations

UPLC–MS/MS: Ultra performance liquid chromatography–tandem mass spectrometry
OTU: Operational taxonomic unit
QC: Quality control
ANOVA: Analysis of variance
LSD: Least significant difference
PCoA: Principal coordinate analysis
PERMANOVA: Permutational multivariate analysis of variance
OPLS-DA: Orthogonal partial least squares discriminant analysis
VIP: Variable importance in the projection
FDR: False discovery rate
T: Treatment
G: Genotype
SRA: Sequence read archive
