# Supplementary material for: Integrated Microbiome and Metabolomic Analysis Reveal Responses of Rhizosphere Bacterial Communities and Root exudate Composition to Drought and Genotype in Rice (Oryza sativa L.)
Source: Rice (N Y). 2023 Apr 11;16:19. doi: 10.1186/s12284-023-00636-1 (PMC10090257; doi:10.1186/s12284-023-00636-1)
Supplement: Supplementary file 2 — Additional file 2: Fig. S1. Rarefaction curves of bacterial communities for the a greenhouse experiment and b soil incubation experiment. Fig. S2. Number of differentially abundant genera a between the two rice genotypes and b between the different watering treatments. Fig. S3. Hierarchical cluster analysis of primary metabolites identified in four groups. Fig. S4. Correlation analysis of all identified metabolites for each root exudate sample. Fig. S5. Principal coordinates analysis (PCoA) of root exudates based on Bray–Curtis distance. Fig. S6. Representative differential metabolites between control and drought treatments for both rice genotypes. Fig. S7. Representative differential metabolites between Nipponbare and Luodao 998 under drought. Fig. S8. Procrustes analysis and Mantel test of the correlation between rhizosphere bacterial communities and root exudates. Fig. S9. Examples of associations between individual differentially abundant genera and differential root exudates. [file 12284_2023_636_MOESM2_ESM.docx]

Supplementary Information

**Integrated microbiome and metabolomic** **analyses reveal responses of rhizosphere bacterial communities and root exudate composition to drought and** **genotype in rice (*Oryza sativa* L.)**

Gege Li^1^, Kexin Wang^1^, Qun Qin^1^, Qi Li^1^, Fei Mo^1^, Vinay Nangia^2^ and Yang Liu^1*^

1. College of Agronomy, Northwest A&F University, Yangling 712100, Shaanxi, China

2. International Center for Agricultural Research in the Dry Areas, Rabat 999055, Morocco

^*^Correspondence: liuyang0328@126.com; yangl@nwafu.edu.cn (Y. Liu)

**Additional file 1:** **Table S1** Effects of drought and genotype on alpha diversity of rhizosphere bacterial communities.

**Additional file 1: Table S2** Effects of drought and genotype on relative abundances (%) of the dominated bacterial phyla.

**Additional file 1: Table S3** Significant tests of drought and genotype on metabolomic properties of root exudates.

**Additional file 2: Fig. S1** Rarefaction curves of bacterial communities for the (a) greenhouse experiment and (b) soil incubation experiment. NI, Nipponbare; LD, Luodao 998; CK, control; D, drought.

**Additional file 2:** **Fig. S2** Number of differentially abundant genera (a) between the two rice genotypes and (b) between the different watering treatments.

**Additional file 2:** **Fig. S3** Hierarchical cluster analysis of primary metabolites identified in four groups. All metabolites are clustered using the Euclidean distance. NI, Nipponbare; LD, Luodao 998; CK, control; D, drought.

**Additional file 2:** **Fig. S4** Correlation analysis of all identified metabolites for each root exudate sample. Asterisks indicate significant correlations (****p* < 0.001; Pearson correlation). NI, Nipponbare; LD, Luodao 998; CK, control; D, drought.

**Additional file 2:** **Fig. S5** Principal coordinates analysis (PCoA) of root exudates based on Bray-Curtis distance.

**Additional file 2: Fig. S6** Representative differential metabolites between control and drought treatments for both rice genotypes. The abundance of metabolites is visualized after log2 transformation. Error bars represent standard deviation.

**Additional file 2:** **Fig. S7** Representative differential metabolites between Nipponbare and Luodao 998 under drought. The abundance of metabolites is visualized after log2 transformation. Error bars represent standard deviation.

**Additional file 2:** **Fig. S8** Procrustes analysis and Mantel test of the correlation between rhizosphere bacterial communities and root exudates. Red and green dots represent rhizosphere bacterial communities and root exudates, respectively.

**Additional file 2: Fig. S9** Examples of associations between individual differentially abundant genera and differential root exudates. The abundance of metabolites is visualized after log2 transformation. The shaded areas represent 95% confidence interval. NI, Nipponbare; LD, Luodao 998; CK, control; D, drought.

**
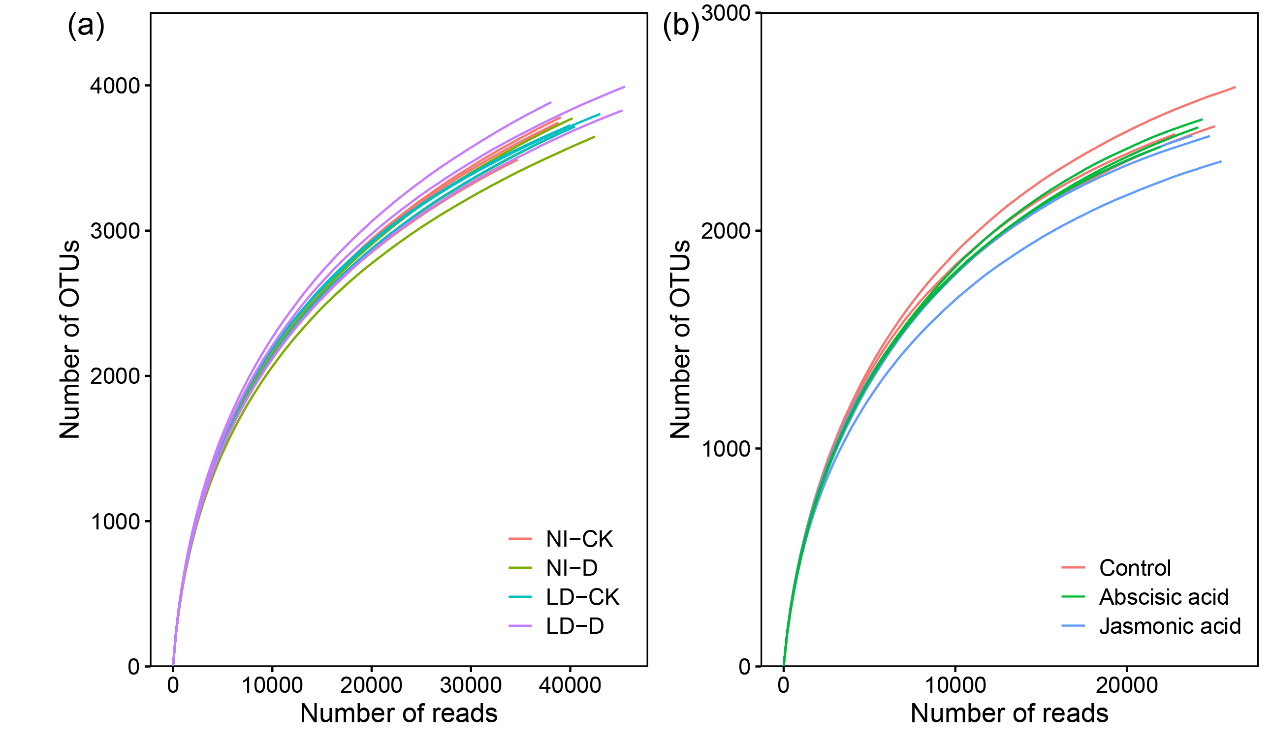
Fig. S1** Rarefaction curves of bacterial communities for the (a) greenhouse experiment and (b) soil incubation experiment. NI, Nipponbare; LD, Luodao 998; CK, control; D, drought.


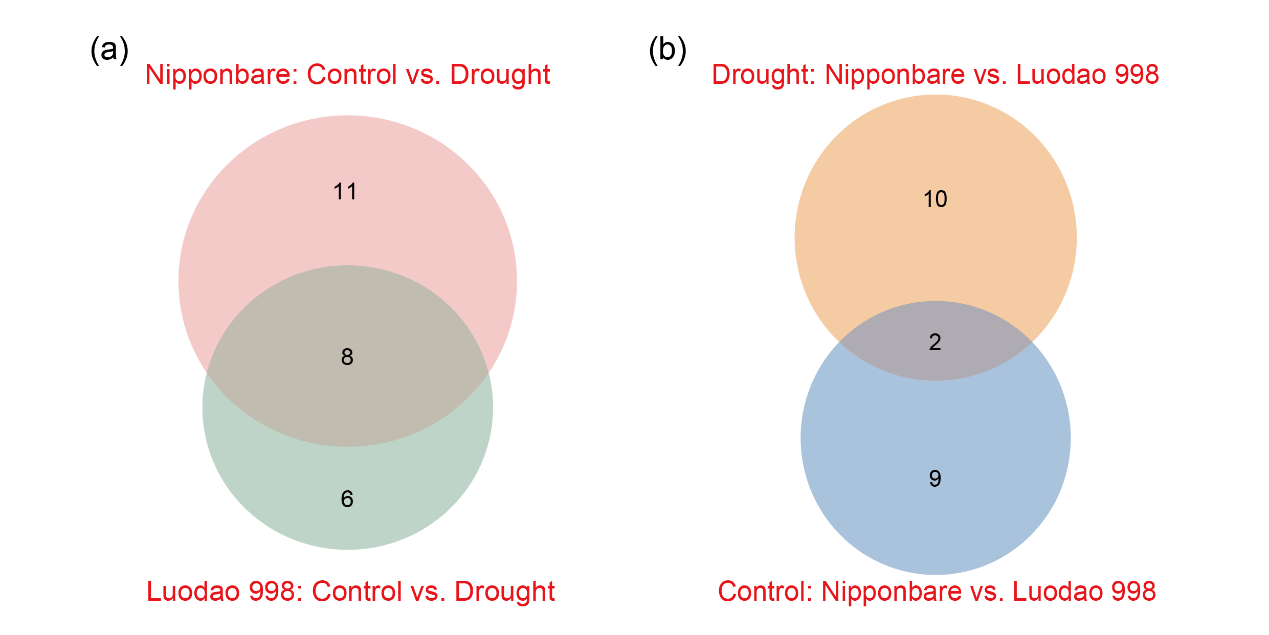


**Fig. S2** Number of differentially abundant genera (a) between the two rice genotypes and (b) between the different watering treatments.


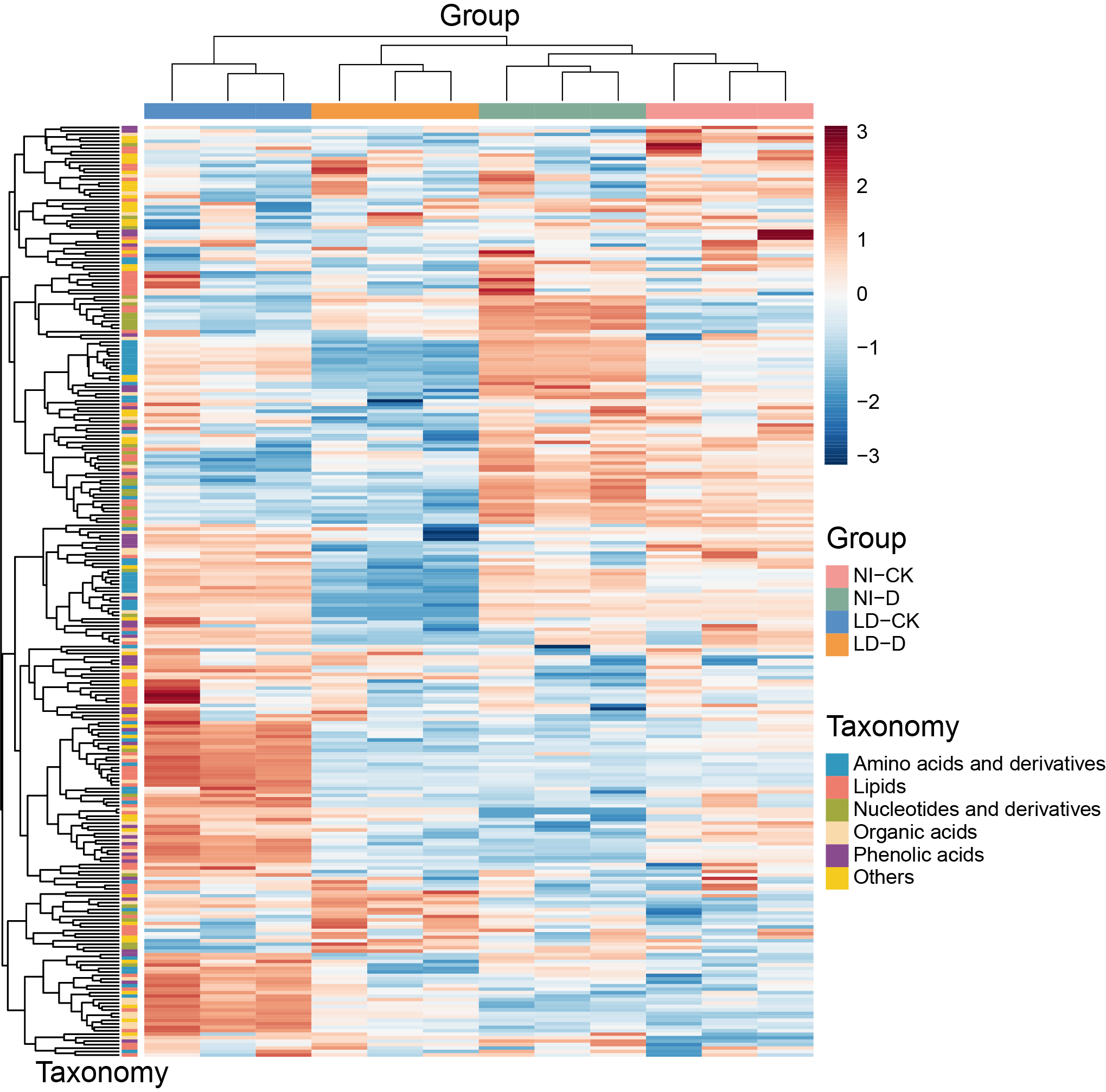


**Fig. S3** Hierarchical cluster analysis of primary metabolites identified in four groups. All metabolites are clustered using the Euclidean distance. NI, Nipponbare; LD, Luodao 998; CK, control; D, drought.


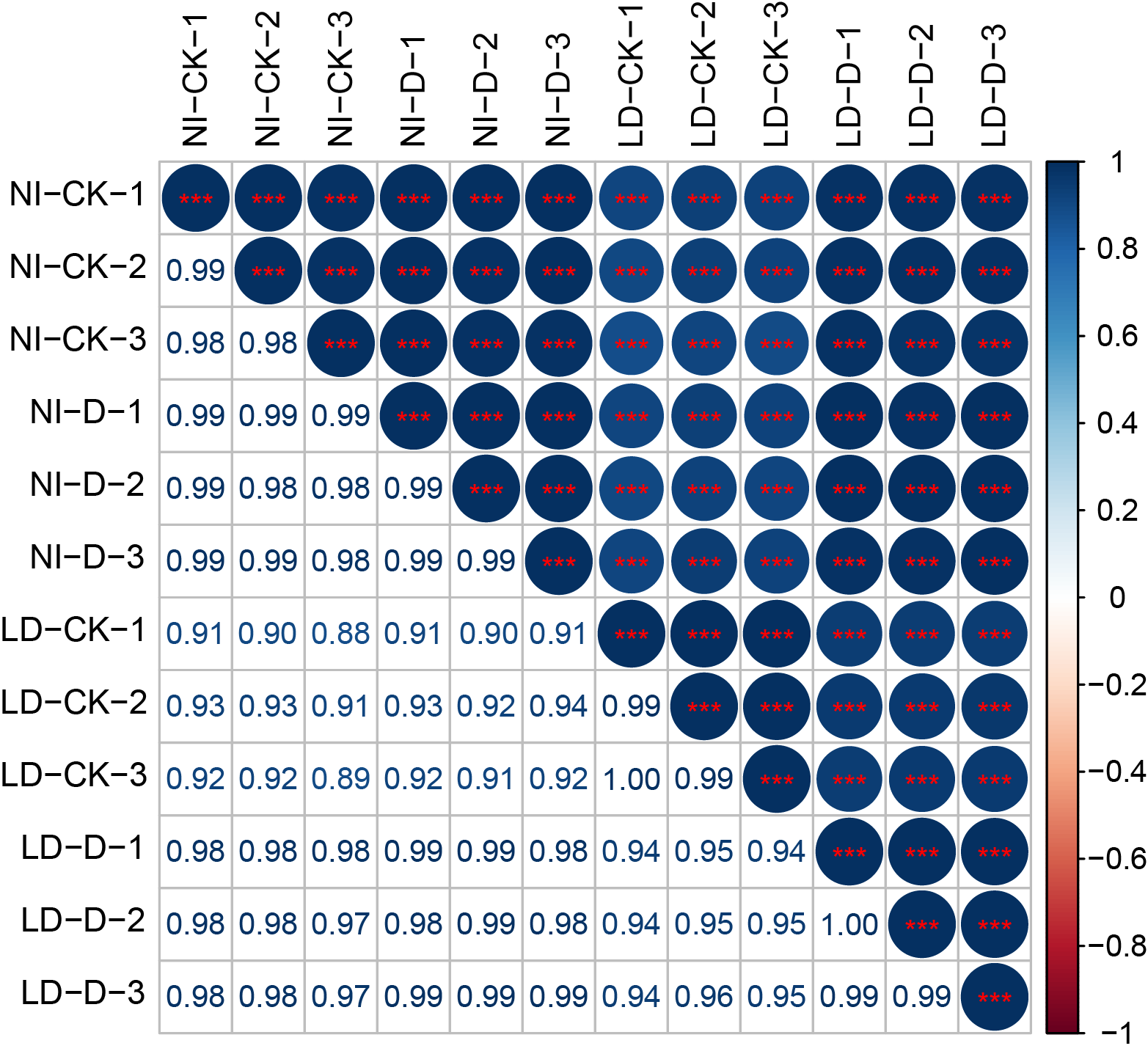


**Fig. S4** Correlation analysis of all identified metabolites for each root exudate sample. Asterisks indicate significant correlations (****p* < 0.001; Pearson correlation). NI, Nipponbare; LD, Luodao 998; CK, control; D, drought.


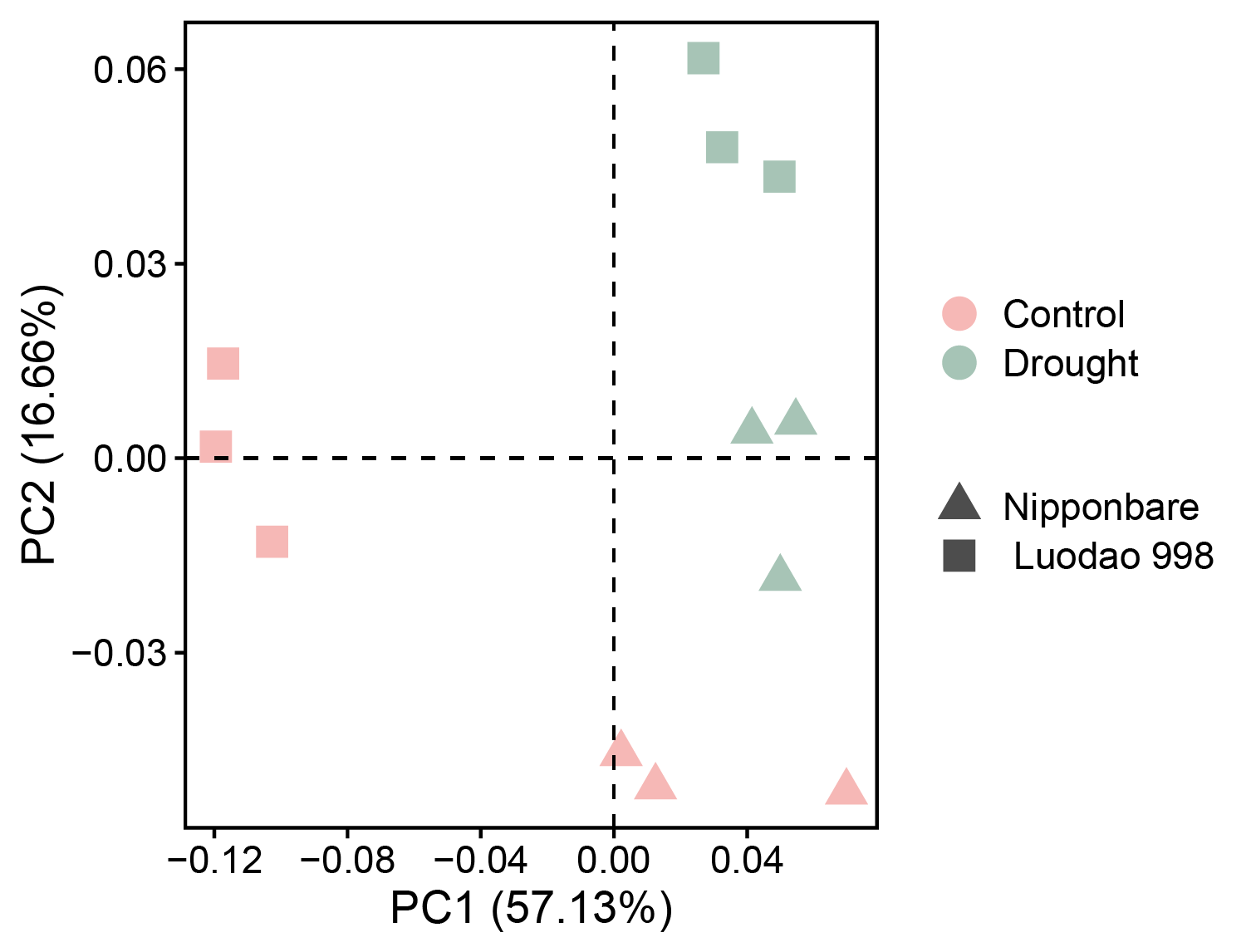


**Fig. S5** Principal coordinates analysis (PCoA) of root exudates based on Bray–Curtis distance.


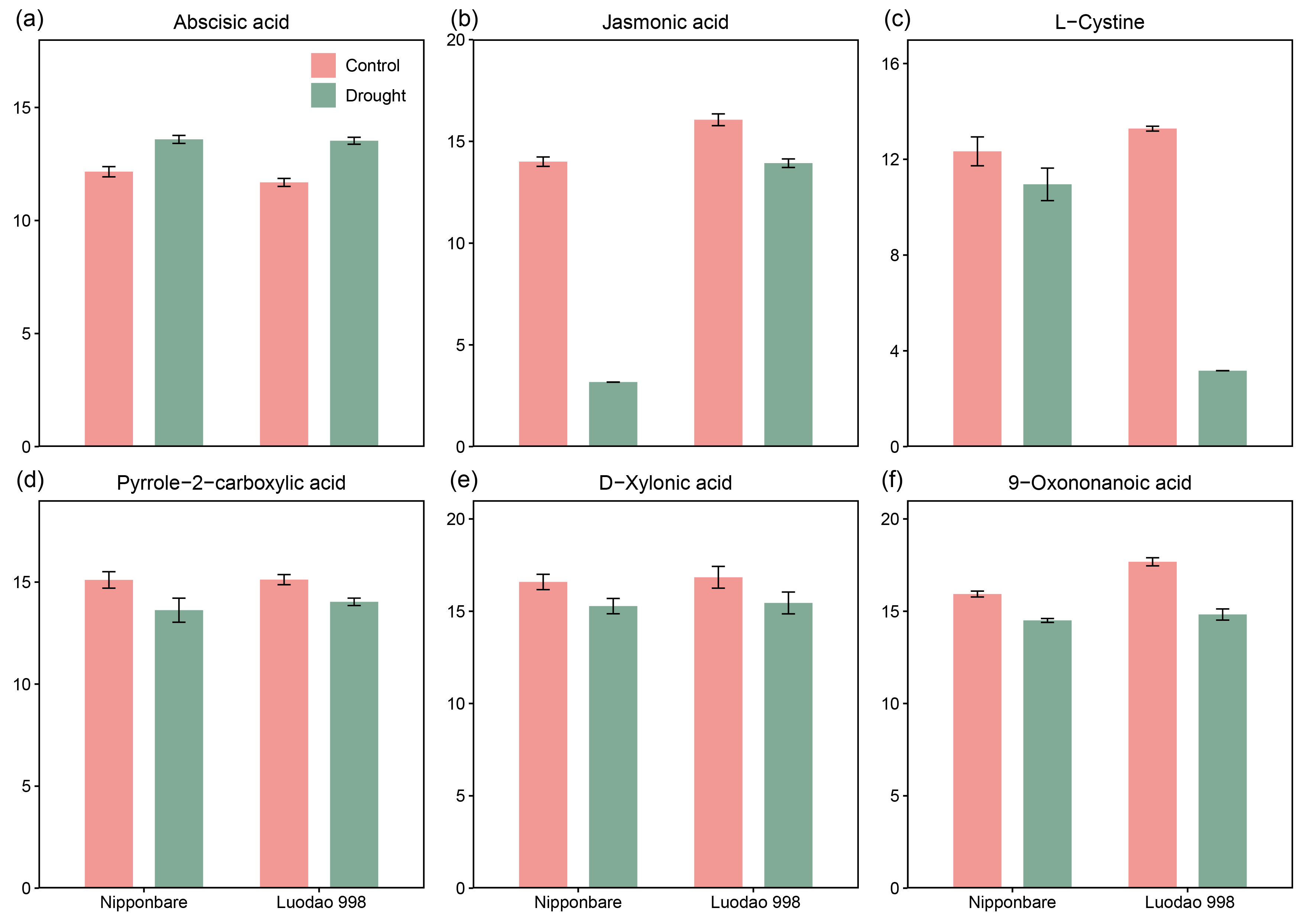


**Fig. S6** Representative differential metabolites between control and drought treatments for both rice genotypes. The abundance of metabolites is visualized after log2 transformation. Error bars represent standard deviation.


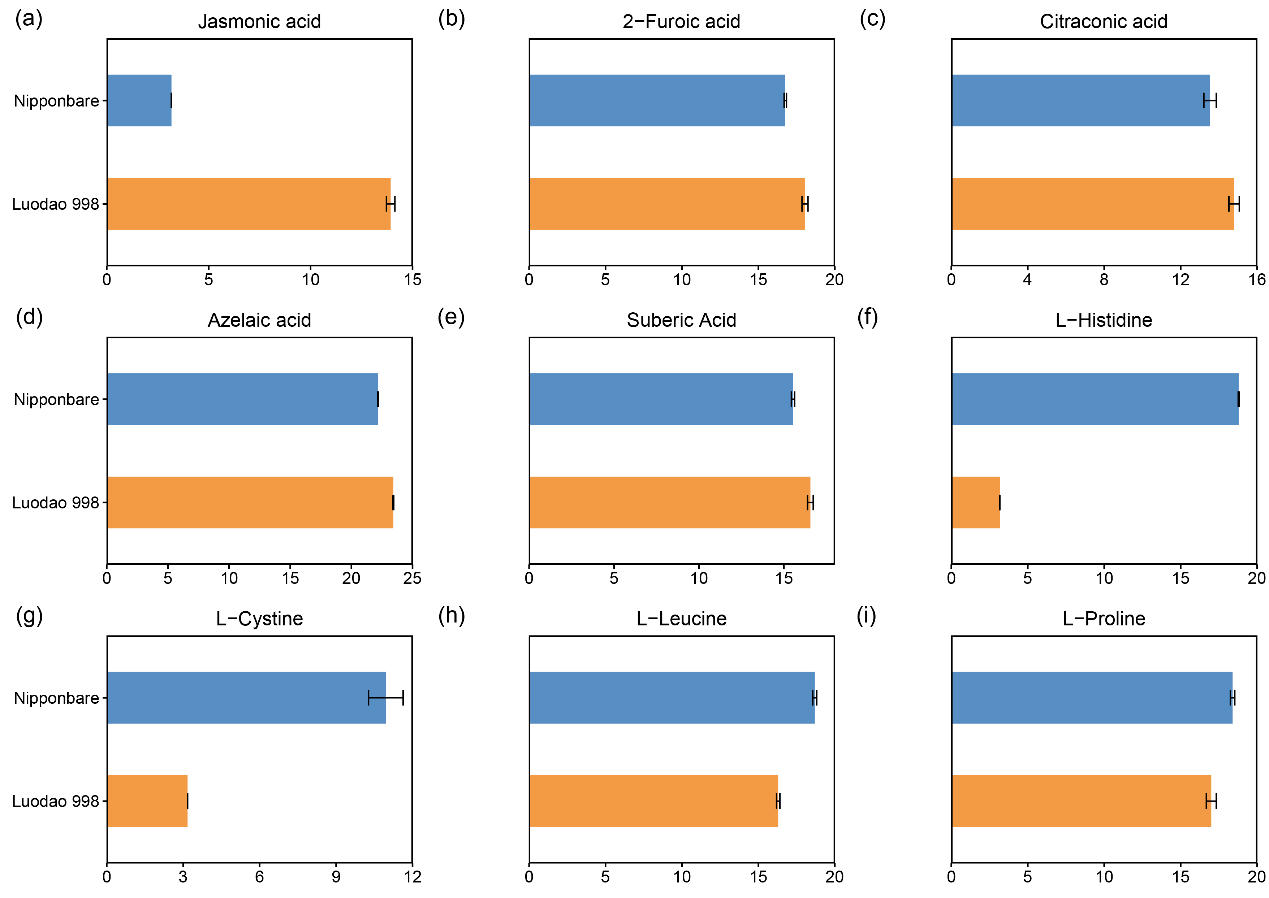


**Fig. S7** Representative differential metabolites between Nipponbare and Luodao 998 under drought. The abundance of metabolites is visualized after log2 transformation. Error bars represent standard deviation.

**
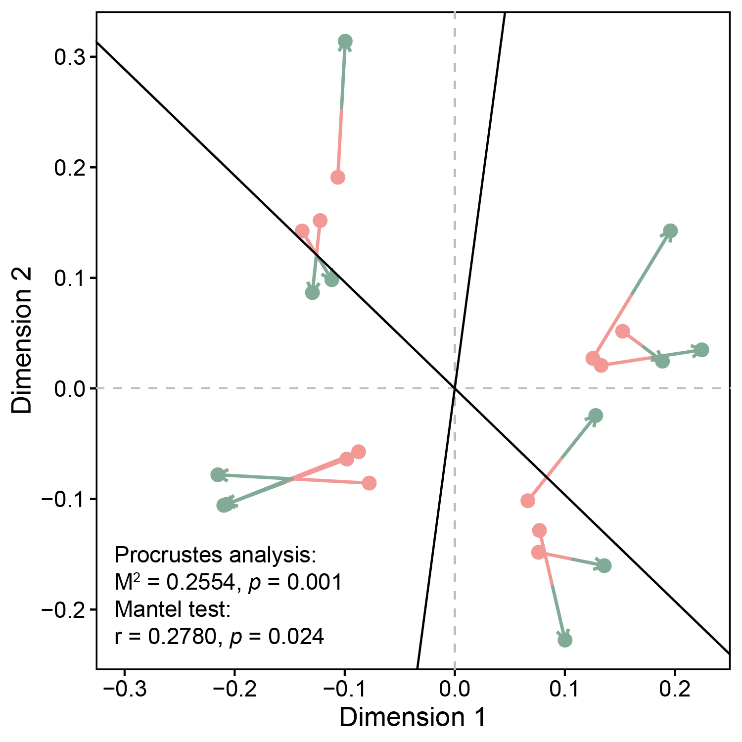
**

**Fig. S8** Procrustes analysis and Mantel test of the correlation between rhizosphere bacterial communities and root exudates. Red and green dots represent rhizosphere bacterial communities and root exudates, respectively.

**
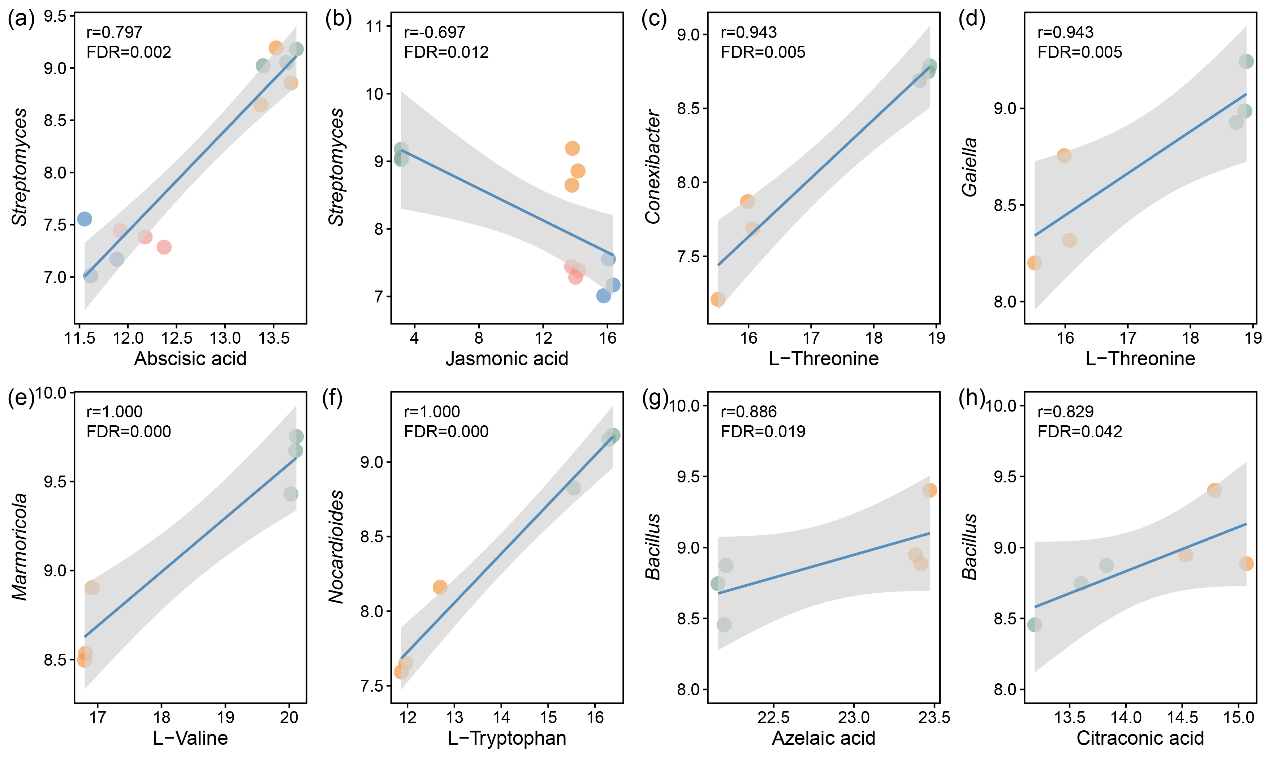
**

**Fig. S9** Examples of associations between individual differentially abundant genera and differential root exudates. The abundance of metabolites is visualized after log2 transformation. The shaded areas represent 95% confidence interval. NI, Nipponbare; LD, Luodao 998; CK, control; D, drought.
